# Developing a checklist for guideline implementation planning: review and synthesis of guideline development and implementation advice

**DOI:** 10.1186/s13012-015-0205-5

**Published:** 2015-02-12

**Authors:** Anna R Gagliardi, Catherine Marshall, Sue Huckson, Roberta James, Val Moore

**Affiliations:** University Health Network, Toronto, Canada; Guideline Adviser and Health Sector Consultant, Waipukurau, New Zealand; Australian and New Zealand Intensive Care Society, Melbourne, Australia; Scottish Intercollegiate Guidelines Network, Edinburgh, Scotland; National Institute for Health and Care Excellence, London, England

**Keywords:** Guidelines, Implementation

## Abstract

**Background:**

Developers, users and others have requested or advocated for guidance on how to plan for, and implement guidelines concurrent to their development given that existing resources are lacking such information. The purpose of this research was to develop a guideline implementation planning checklist.

**Methods:**

Documents that described or evaluated the processes of planning or undertaking implementation were identified in several publications that had systematically identified such resources, and by searching medical literature databases (MEDLINE, EMBASE). Data that described implementation planning; how to develop guideline versions or tools that would support user implementation; and options and mechanisms for disseminating or implementing guidelines were independently extracted from eligible documents by the principal investigator and a trained research assistant. Data were integrated to create a unique list of guideline implementation planning processes and considerations.

**Results:**

Thirty-five documents were eligible. Of these, 16 (45.7%) provided sparse information on implementation planning, 25 (71.4%) mentioned different versions or tools for implementation, and 30 (85.7%) listed options for dissemination or implementation. None provided instructions for operationalizing implementation strategies. Data were integrated into a multi-item Guideline Implementation Planning Checklist including considerations for implementation planning (12), development of implementation tools (8), types of implementation tools (12), and options for dissemination (11) and implementation (12).

**Conclusions:**

Developers or users can apply the Guideline Implementation Planning Checklist to prepare for and/or undertake guideline implementation. Further development of the checklist is warranted to elaborate on all components. In ongoing research, we will consult with the international guideline community to do so. At the same time, guideline implementation is complex, so developers and users would benefit from training, and by including knowledge translation experts and brokers on implementation planning committees.

**Electronic supplementary material:**

The online version of this article (doi:10.1186/s13012-015-0205-5) contains supplementary material, which is available to authorized users.

## Background

Guidelines are documents that synthesize current evidence on how to most effectively organize and deliver health services for a given condition [1]. They inform healthcare decision-making and can serve as the basis for policy, planning, evaluation, and quality improvement. When newly developed, or when evaluation identifies suboptimal compliance, efforts are needed to promote awareness, acceptance, adoption, and adherence to guidelines. Such efforts include dissemination (posting on a web site, publishing in a journal, presenting information at a meeting) or implementation (purposeful strategies that employ educational, social, organizational, financial or technological means of promoting guideline use) [2]. However, guidelines are not always translated into policy or practice [3,4]. A systematic review of studies in which guideline use was evaluated revealed that adoption and adherence were low even when awareness of, and agreement with guidelines among target users was high [5]. This suggests that dissemination efforts are suitable for sharing guidelines but lack of, or poor implementation limits guideline use.

Guideline implementation is challenged by many issues. The effectiveness of most implementation strategies is small and inconsistent, limiting their impact on guideline adoption [6]. A variety of contextual factors at the individual, institutional and system level often co-exist and pose additional challenges to guideline implementation and use [7]. Promotional efforts by guideline developers may be constrained by lack of resources so implementation is often the responsibility of target users [8,9]. Regardless of whether implementation is undertaken by developers or users, implementation is further complicated by two key factors. One, while instruments exist to assess barriers of guideline use, or organizational capacity or readiness to adopt guidelines, these do not reliably identify the most appropriate implementation strategy for a given guideline [10,11]. Two, implementation planning most often occurs upon guideline completion. Implementation could be more successful if planning were concurrent rather than consecutive to guideline development so that the recommendations were clear and useable, target users were primed for adoption, and their needs and preferences, and insight on contextual factors could inform implementation planning [12]. This may also reduce the time required for guidelines to be adopted into policy or practice by avoiding a lengthy waiting period from guideline completion to implementation planning, and actual execution of implementation activities.

Developers, users and others have requested or advocated for guidance on how to plan and implement guidelines [13-15]. While taxonomies [16,17] and models [18] of guideline implementation strategies and approaches are available, and considerable research has synthesized primary studies to report on the effectiveness of guideline implementation strategies [19], these sources do not offer guidance on how to plan for the implementation of guidelines. Schunemann et al. recently issued a checklist for developing guidelines, however, it focused largely on planning for, and undertaking guideline development, and addressed implementation in brief following completion of guideline development [20]. Developers and users would benefit from information on the steps and considerations for guideline implementation planning. This would supplement the Schunemann et al. checklist [20] and support guideline implementation and use among developers and users, leading to improved use of guidelines in health care decision-making. The purpose of this research was to develop a guideline implementation planning checklist.

## Methods

### Approach

A working group representing guideline developers, implementers and researchers was assembled and met by teleconference on January 14, 2014 to discuss the type of resource that would be of greatest use to those implementing guidelines. The working group was drawn from members of the Guidelines International Network (G-I-N) Implementation Working Group. G-I-N is a global network comprised of 100 organizations and 131 individual members representing 48 countries from all continents (www.g-i-n.net). The intent was to create a checklist rather than a manual with detailed instructions and templates which would require considerable time and resources to initially develop, and for each required update. Furthermore, implementation must be tailored to context so a single manual could not provide advice that is broadly relevant. Instead, a checklist could be more quickly developed given our limited resources for doing so; draw upon useful resources that already existed; and provide quick, practical advice that could be broadly applied, and easily updated as needed. We examined the content of documents that described the process of planning for guideline implementation, and organized and summarized the information into a checklist. Ethical approval was not required as data were collected from publicly available resources. Although not a traditional systematic review, the Preferred Reporting Items for Systematic Reviews and Meta-Analyses (PRISMA) criteria guided reporting of the methods and findings (Additional file 1: Table S1) [21].

### Sampling

Documents were identified in the reference lists of recently published studies that had systematically identified manuals and other relevant documents describing guideline development [20,22,23]. The studies described in these publications, while relevant, had focused on guideline development and may not have been comprehensive. Therefore, we searched OVID MEDLINE and EMBASE from 2004 to 2013 on January 25, 2014 (Additional file 2: Table S2) to identify additional studies, reports or manuals that focused on guideline implementation, or any guideline development manuals that may have been missed in previous studies. Search terms were informed by research that generated search strategies for implementation topics which optimized sensitivity and specificity [24,25]. The principal investigator and a trained research assistant independently reviewed the search results to assess eligibility. Eligible documents included English-language studies, reports or instructional manuals that were publicly available or published in a peer-reviewed journal, and described or evaluated the processes of planning or undertaking implementation. Those published from 2004 to 2013 were included so that recommended processes would be reasonably current. Guidelines that described clinical recommendations were excluded, as were single studies or systematic reviews evaluating the effectiveness of implementation strategies since our intent was to describe implementation planning and not to evaluate interventions. Manuals prepared by for-profit health care delivery or guideline development organizations, and meeting abstracts were not eligible. All items considered eligible by at least one reviewer were retrieved for full text screening during data extraction.

### Data collection

Data were first extracted by the principal investigator. The full text of each eligible document was perused to identify, extract and tabulate any information that referred to or described implementation planning (defined as instructions for when and how to plan and prepare for implementation); implementation tools (defined as instructions for developing guideline content, versions or tools that support implementation); and dissemination and implementation (defined as strategies and instructions for distributing, sharing, promoting, and applying guideline recommendations). The location of the information within each document was also noted. The full extent of information available in each source was extracted, though not necessarily verbatim, except when it was too lengthy or detailed to duplicate. A research assistant checked extracted data against the original source to ensure that all relevant information was accurately and fully retrieved from each item.

### Data analysis

The total number of eligible manuals produced by different types of organizations, and the number of manuals addressing different aspects of implementation were summarized. Extracted data were integrated to create a step-by-step list of unique processes and considerations for implementing guidelines from planning to execution.

## Results

**Figure 1:**
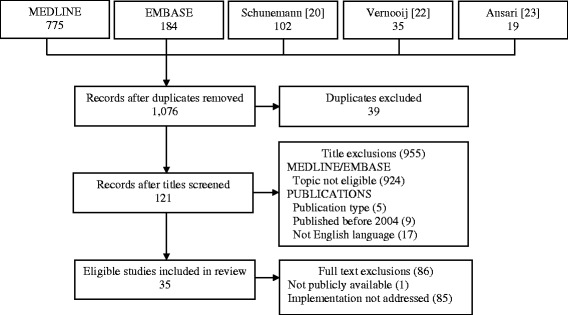
**PRISMA flow diagram of search results.**

The screening process and results are outlined in Figure 1. The Schunemann [20], Vernooij [22] and Ansari [23] articles referenced 102, 35, and 19 relevant documents, respectively. The MEDLINE and EMBASE searches together retrieved 959 articles. Following the removal of duplicates, 1,076 titles were screened. Of these, 955 titles were eliminated (924 ineligible topic, 5 ineligible publication type, 9 published before 2004, and 17 not English language). Screening of the full text of 121 remaining items eliminated a further 86 (1 not publicly available, 85 implementation not addressed). As a result, 35 unique documents were eligible for review. The majority of these reflected guideline development methods used by professional societies or foundations (*n* = 22) or government agencies (*n* = 10) plus one each from a not-for-profit agency, academic organization, and a peer-reviewed research publication on guideline implementation.

Data extracted from eligible documents are presented in Additional file 3: Table S3. Of the 35 documents, 16 (45.7%) provided information on implementation planning, 25 (71.4%) mentioned different versions or tools that were meant to support implementation, and 30 (85.7%) mentioned strategies that could be used to disseminate or implement guidelines or guideline implementation tools. Most resources offered sparse guidance for implementation planning, choosing and operationalizing dissemination and implementation strategies, or developing alternate versions or tools that supported implementation.

Data across all sources were integrated to create a list of unique steps and considerations for guideline implementation planning (Table 1). Key implementation planning actions included assembling an implementation planning team that may or may not be part of the guideline development group; assembling resources for implementation; auditing baseline practice; assessing barriers and interacting with stakeholders to gather contextual information that would inform the selection of dissemination and implementation strategies, and the development of guideline implementation tools; and the preparation of an implementation plan specifying strategies, roles, responsibilities, timelines, and measures by which to evaluate the implementation process and outcomes. A number of options for adjunct products to support user implementation were identified including alternate versions and implementation tools, though only one resource provided information about processes that could be used to develop implementation tools. Several resources offered similar options for dissemination and implementation. Frequently mentioned dissemination options included posting guidelines and implementation tools on a web site and publishing them in journals. The most frequently mentioned implementation options included educational meetings, and audit and feedback.

**Table 1:** **Guideline implementation planning checklist**

**Steps or considerations**	**References**
*Implementation planning*—instructions for when and how to plan and prepare for implementation	
Implementation should be considered at the beginning, and throughout the guideline development process	[26-29]
Form an implementation team from the start that includes stakeholders (patient groups, end users, champions, relevant organizations and agencies, policy-makers) and one or more knowledge translation experts	[26-28,30-33]
Identify and assemble resources for implementation	[27,34]
Audit current practice as a baseline needs assessment	[32]
Consider or assess barriers of guideline implementation and use (patient, professional, organizational, system, economic, political, social/cultural), and stakeholder needs and preferences through literature review, observation, focus groups, interviews or survey	[27,30-39]
Consider dissemination and/or implementation on a recommendation-by-recommendation basis rather than for entire guideline	[33]
Determine the dissemination and implementation strategies that are effective and best suited to address identified needs and barriers	[27,30-36,40]
Determine what implementation tools will be developed based on: guideline scope; guideline recommendations; identified knowledge gaps or baseline audit; interviews or focus groups with guideline users	[30,35,40,41]
Develop an implementation plan describing dissemination and implementation strategies and tools, roles and responsibilities, milestones, time frames, and implementation measures	[26-28,32,38,42]
Consider pilot testing the implementation strategy on a small scale and adjust as needed after the pilot test and on an ongoing basis	[32,34,38]
Continue to engage stakeholders with outreach and education throughout the guideline development process	[27,30-33,40]
Ensure guideline recommendations are implementable and can be integrated in computer decision support systems	[35,41]
*Implementation tools*—instructions for developing guideline content, versions, or tools that support implementation	
Research potential designs for type(s) of tools selected	[30]
Identify resources that will be needed	[30]
Present draft tool to guideline development group	[30]
Refine the tool with feedback, and several iterations may be needed	[30]
Test tool usability with clinician or patient interviews or focus groups	[30]
Feedback is used to refine the tool	[30]
Final version is reviewed by the guideline development group	[30]
Implementation tools are published at the same time as the guideline	[26]
*Potential implementation tools include:*	
Versions in different languages	[27,28,43]
Versions in different formats (mobile devices, pocket guide, wall poster)	[26,29-31,44-47]
Summary versions (short version, recommendations only, evidence only)	[26,28,29,32,35,37,38,42-44,46-51]
Patient or plain language version	[26,27,35,43,46,48,49]
Point-of-care tools (algorithms, checklists, decision aids)	[27,28,35,36,38,48-50,52]
Electronic medical record/computer decision support system integration	[30]
Implementation plan (recommended strategies, barriers specific to the guideline and its recommendations, instructions)	[28,36,43,48,53,54]
Teaching aids (slide set, case examples, meeting agenda)	[27,42,47,49,52,53]
Patient and caregiver resources	[28,32,35,36,45,48,50,52]
Resource planning guide (human, infrastructure, technological capacity needed to implement and apply the recommendations)	[36,48]
Costing tools (spreadsheet, report templates)	[26,53]
Evaluation plan (instructions, measures, data collection instruments)	[26-28,32,35,36,39,45,48,50,53]
*Dissemination and implementation*—strategies for distributing, sharing, promoting and applying guideline recommendations	
*Dissemination options include:*	
Web site (guideline, implementation tools, accredited CPD modules)	[26,27,29,32,35,38,43-49,51,55-58]
Journal publications (which can link to online material)	[26,27,29,30,35,37,42-49,51,55-57,59,60]
Press release	[31,35,47,49]
Mass media campaign	[27,30,32-34,41]
Email distribution	[35,44,49]
Podcast or webinar	[35,45]
Register with AHRQ Guideline Clearinghouse and G-I-N Library	[35]
Partnerships (national organizations, networks)	[26,31,45]
Endorsement by specialty society	[31,45]
Marketing strategies	[31]
Traditional arts	[31]
*Implementation options include:*	
Printed educational material	[31-34,36,40,41]
Educational meetings (conferences, workshops, CPD)	[26,30-36,39-42,44,46,60]
Educational outreach/Academic detailing	[32-34,36,40,41,60]
Local opinion leaders	[32-34,36,40,41,60]
Audit and feedback	[32-34,36,40-42,60]
Reminders	[32,33,36,39-41,60]
Multi-faceted interventions	[32,36]
Patient-mediated interventions (educational material, decision support tools, mass media campaign, reminders)	[32-43,40]
Organizational interventions (revision of professional roles or teams, leadership engagement)	[33,34,40]
Financial incentives or penalties	[32,33,41]
Computer decision support systems	[33,41]
Regulatory interventions/accreditation	[33,41,42]

## Discussion

We developed a Guideline Implementation Planning Checklist by conducting a comprehensive review of existing resources. This can be applied by guideline developers or users to prepare for and/or undertake implementation. Information was variable across documents, and, in general, quite sparse. In large part this was due to the fact that most resources focused on guideline development, and few were dedicated to guideline implementation. As a result, the Guideline Implementation Planning Checklist is brief. However, it provides developers or users with a framework for implementation planning that coincides with guideline development, can be adapted to context-specific factors and therefore may be broadly applicable, can be employed by guideline developers or users so its application is flexible and, as a synthesis of available information, it is more detailed than existing resources.

This research is limited in that our search strategy or screening may have failed to retrieve or identify all relevant resources, stringency of screening criteria may have excluded otherwise eligible documents, and we may not have accurately extracted or synthesized data. To mitigate these issues we performed a systematic search of previous comprehensive syntheses of guideline development and implementation documents, and of indexed databases for published healthcare research; and data extraction and synthesis was performed independently by two individuals which enhances reliability of the findings.

As noted, the content of the Guideline Implementation Planning Checklist is limited by the content of existing documents from which it was compiled which, in turn, is influenced by limited evidence for these processes. With respect to planning for implementation, there is no evidence on the effectiveness of planning steps or considerations, nor is it practical that such studies would be conducted. For example, the impact of forming an implementation team or developing an implementation plan on the conduct and outcomes of implementation planning is a logistical consideration, and would likely not be a priority for research funders. Evidence is mixed on the impact of assessing barriers as a means of choosing and tailoring interventions. For example, a Cochrane systematic review of 25 studies found that interventions tailored based on identified barriers were more likely to improve professional practice compared with no intervention or with dissemination of guidelines, however, methods used to identify barriers and tailor interventions were not described in eligible studies so how best to do this remains unclear [61]. Another systematic review of 63 studies across which the same instrument was used to assess barriers found that, although it identified numerous barriers, this was not reflected in the tailoring of implementation strategies, and was not associated with evidence adoption [62]. While several eligible documents recommended engaging stakeholders throughout the implementation planning process, few details were offered. Knowledge brokers can function as liaisons between those generating and applying evidence. Qualitative analysis of a randomized controlled trial revealed numerous ways in which knowledge brokers enhanced the capacity of public health officials to practice evidence-informed decision-making, however, the trial found their impact to be equivalent to that of evidence summaries so the authors recommended ongoing research to optimize their role [63]. The principles and practices of action research or integrated knowledge translation could be used to elaborate on how knowledge brokers could establish and nurture partnerships with stakeholders [64,65]. While evidence underlying the planning strategies identified in eligible documents may be limited, wide consultation with, and the consensus of guideline developers and implementers could be used to enhance the implementation planning component of the Guideline Implementation Planning Checklist until further evidence becomes available.

With respect to developing guideline implementation tools, this content could be expanded based on recent research in which Gagliardi et al. engaged the international guideline community to establish a 12-item framework of the desirable features of implementation tools, and identified 11 broad methodological steps, each with several sub-steps and considerations for developing implementation tools [66]. This knowledge can be applied to assess, and endorse or adapt existing implementation tools, or develop new implementation tools. Addition of these methods to the Guideline Implementation Planning Checklist may further enhance its relevance. However, further research is needed to develop guides or templates specific to different types of implementation tools.

With respect to dissemination, the list of options available in eligible documents and compiled in the Guideline Implementation Planning Checklist matches several items within the Professional Interventions domain of taxonomies of methods that have been used to implement guidelines including distribution and advertising of guideline material through mailing and mass media [16,17]. However, neither the taxonomies nor eligible documents comment on the comprehensiveness of the list of dissemination options, or on their effectiveness for achieving awareness and use of guidelines among different target audiences. In fact, the comparative effectiveness of such strategies is unclear. A Cochrane systematic review of printed educational materials, which included guidelines, reviewed data from 45 studies and concluded that, when used alone, such material can have a small impact on professional practice [67]. Another Cochrane systematic review of eight studies found that mass mailing of printed bulletins summarizing evidence may improve practice when there is a single, clear message, if the change is relatively simple to accomplish, and there is growing awareness by users of the evidence that a change in practice is required [68]. A systematic review of 42 articles reporting 38 dissemination studies by the Agency for Healthcare Research and Quality (AHRQ) similarly identified little comparative effectiveness research upon which to base the selection of dissemination strategies [69]. While evidence was limited, the AHRQ review found that reach strategies (dissemination using regular mail, email, or social or mass media) were as effective as ability strategies (providing users with additional resources or tools), and that multicomponent strategies involving a combination of research, ability and motivational strategies (opinion leaders, champions, etc.) appeared to be more effective than using one strategy alone, particularly for guideline adherence. Thus, some evidence exists to show that various means of dissemination can reach target audiences. This is further confirmed by a systematic review of studies that evaluated guideline implementation efforts which found that dissemination of guidelines through web sites, journal publications or other means resulted in awareness of, and agreement with guidelines among target users [5]. However, the same systematic review revealed that target users struggled with adoption, and other research involving interviews and focus groups with clinicians similarly revealed that they were frustrated with and uncertain about how to implement guidelines [14]. Therefore, guideline developers and users require implementation advice.

Eligible documents offered several implementation options but the compiled list was not as comprehensive as the options in published taxonomies of implementation strategies [16,17]. Moreover, the implementation strategies most frequently recommended in eligible documents were educational meetings and audit and feedback. Considerable evidence suggests that the impact of educational meetings is small and, while perhaps necessary as an initial means of raising awareness and engaging stakeholders, may need to be accompanied by other strategies that are informed by a contextual needs assessment [70]. Audit and feedback has also been investigated in a large number of trials and can achieve small to moderate impact when it is delivered by a supervisor or respected colleague, presented frequently, features both specific goals and action plans, aims to decrease the targeted behavior, baseline performance is low, and recipients are non-physicians [71]. In future research, the Guideline Implementation Planning Checklist could be improved by referring users to taxonomies of dissemination and implementation strategies [16,17], and systematic reviews that evaluate their effectiveness [6].

Given the conflicting evidence on the effectiveness of various implementation strategies, and the nuanced application of these strategies which requires contextual analysis and tailoring of strategies, it simply may not be possible to capture those details in a checklist or, indeed, even in a more detailed instructional manual such as those from which data were extracted. Therefore, the Guideline Implementation Planning Checklist could be applied in conjunction with additional strategies to develop implementation capacity among guideline developers and users. For example, a few eligible resources mentioned that the implementation team should include one or more knowledge translation experts. This may refer to researchers, managers or clinicians with expertise or experience in guideline implementation. It may also refer to knowledge brokers, who would liaise with both the guideline development or implementation group, and with target stakeholders to plan, develop and/or apply the most feasible and appropriate implementation strategies [72]. Alternatively, individuals charged with developing or implementing guidelines may require implementation training. Knowledge translation training programs have been established in the United States, United Kingdom and Canada [73-75]. Gagliardi et al. conducted a systematic review to generate guidance on how to develop a mentorship program for developing implementation capacity [76]. Through the G-I-N Implementation Working Group, we are pilot-testing the use of remote coaching by implementation experts as a means of providing guideline developers and implementers with guidance on an as-needed basis during the course of their initiatives.

In summary, this research identified that many of the recommended methods for planning guideline implementation may be intuitive but are not supported by evidence, and that lists of suggested options for dissemination and implementation are incomplete and lack descriptions of, or references to supporting evidence which would help developers or implementers choose which to apply. Further research is needed to generate evidence on how to assess barriers and use that information for implementation planning; describe the role of knowledge brokers or other knowledge translation experts in implementation planning; create guides or templates for developing specific types of implementation tools; and identify strategies that may be needed by developers and implementers in conjunction with the Guideline Implementation Planning Checklist to better plan guideline implementation. In ongoing research we will update the Guideline Implementation Planning Checklist by including references or links to relevant resources such as taxonomies of dissemination and implementation strategies, and systematic reviews that describe the effectiveness of those strategies. We will also consult with international guideline developers, implementers, and users to elaborate and improve the methods included in the Guideline Implementation Planning Checklist based on their needs and experiences. We may also conduct research in partnership with guideline developers, implementers and users to evaluate either concurrently or retrospectively their use of the Guideline Implementation Planning Checklist as a means of further refining its components.

## Conclusions

Developers or users can apply the Guideline Implementation Planning Checklist to prepare for and/or undertake guideline implementation. Further development of the checklist is warranted to elaborate on all components. In ongoing research we will consult with the international guideline community to do so. At the same time, guideline implementation is complex, so developers and users would benefit from training, and by including knowledge translation experts and brokers on implementation planning committees.

## Additional files

Additional file 1: Table S1. PRISMA checklist. Additional file 2: Table S2. Search strategy (from MEDLINE). Additional file 3: Table S3. Implementation guidance extracted from eligible resources.

## References

[1] Weisz G, Cambrosio A, Keating P, Knaapen L, Schlich T, Tournay VJ (2007). The emergence of clinical practice guidelines. Milbank Q.

[2] McCormack L, Sheridan S, Lewis M, Boudewyns V, Melvin CL, Kistler C (2013). Communication and dissemination strategies to facilitate the use of health-related evidence. Evid Rep Technol Assess..

[3] McGlynn EA, Asch SM, Adams J, Keesey J, Hicks J, DeCristofaro A (2003). The quality of health care delivered to adults in the United States. NEJM.

[4] Sheldon TA, Cullum N, Dawson D, Lankshear A, Lowson K, Watt I (2004). What’s the evidence that NICE guidance has been implemented? Results from a national evaluation using time series analysis, audit of patients’ notes, and interviews. BMJ.

[5] Mickan S, Burls A, Glasziou P (2011). Patterns of ‘leakage’ in the utilization of clinical guidelines: a systematic review. Postgrad Med J.

[6] Grimshaw JM, Thomas RE, MacLennan G, Fraser C, Ramsay CR, Vale L (2004). Effectiveness and efficiency of guideline dissemination and implementation strategies. Health Technol Assess.

[7] Francke AL, Smit MC, de Veer AJE, Mistiaen P (2008). Factors influencing the implementation of clinical guidelines for health care professionals. BMC Med Inform Dec Mak..

[8] Kryworuchko J, Stacey D, Bai N, Graham ID (2009). Twelve years of clinical practice guideline development, dissemination and evaluation in Canada (1994 to 2005). Implement Sci..

[9] Lavis JN, Oxman AD, Moynihan R, Paulsen EJ (2008). Evidence-informed health policy—interviews with the directors of organizations that support the use of research evidence. Implement Sci..

[10] Simpson KM, Porter K, McConnell ES, Colon-Emeric C, Daily KA, Stalzer A (2013). Tool for evaluating research implementation challenges. Implement Sci..

[11] Helfrich CD, Li YF, Sharp ND, Sales AE (2009). Organizational readiness to change assessment (ORCA). Implement Sci..

[12] Gagliardi AR, Brouwers MC (2012). Integrating guideline development and implementation: analysis of guideline development manual instructions for generating implementation advice. Implement Sci..

[13] Gagliardi AR. ‘More bang for the buck’: exploring optimal approaches for guideline implementation through interviews with international developers. BMC Health Serv Res. 2012;12:404. 10.1186/1472-6963-12-404 PMC3561165 23153052

[14] McKillop A, Crisp J, Walsh K (2012). Practice guidelines need to address the ‘how’ and the ‘what’ of implementation. Primary Health Care Res Develop.

[15] Pronovost PJ (2013). Enhancing physicians’ use of clinical guidelines. JAMA.

[16] Mazza D, Bairstow P, Buchan H, Chakraborty SP, Van Hecke O, Grech C (2013). Refining a taxonomy of guideline implementation. Implement Sci..

[17] Effective Practice and Organisation of Care EPOC: *EPOC Resources for review authors*. Oslo: Norwegian Knowledge Centre for the Health Services, 2013. [Retrieved from: http://epocoslo.cochrane.org/epoc-specific-resources-review-authors]

[18] Graham ID, Logan J, Harrison MB, Straus SE, Tetroe J, Caswell W (2006). Lost in knowledge translation: time for a map?. J Contin Educ Health Prof.

[19] Prior M, Guerin M, Grimmer-Somers K (2008). The effectiveness of clinical guideline implementation strategies—a synthesis of systematic review findings. J Eval Clin Pract..

[20] Schunemann HJ, Wiercioch W, Etxeandia I, Falavigna M, Santesso N, Mustafa R, et al. Guidelines 2.0: systematic development of a comprehensive checklist for a successful guideline enterprise. CMAJ. 2014;186(3):E123–42. 10.1503/cmaj.131237 PMC3928232 24344144

[21] Moher D, Liberati A, Tetzlaff J, Altman DG, The PRISMA Group (2009). Preferred reporting items for systematic reviews and meta-analyses: the PRISMA statement. BMJ..

[22] Vernooij RWM, Sanabria AJ, Sola I, Alonso-Coello P, Martinez Garcia L (2014). Guidance for updating clinical practice guidelines: a systematic review of methodological handbooks. Implement Sci..

[23] Ansari S, Rashidian A (2012). Guidelines for guidelines: Are they up to the task?. PLoS One.

[24] McKibbon KA, Lokker C, Wilczynski NL, Cilisak D, Dobbins M, Davis DA (2010). A cross-sectional study of the number and frequency of terms used to refer to knowledge translation in a body of health literature in 2006: a Tower of Babel?. Implement Sci..

[25] McKibbon KA, Lokker C, Wilczynski NL, Haynes RB, Ciliska D, Dobbins M (2012). Search filters can find some but not all knowledge translation articles in MEDLINE: an analytic survey. J Clin Epi..

[26] National Institute for Health and Care Excellence. The guidelines manual. Chapter 13. Implementation support. UK: NICE; 2012. 27905714

[27] World Health Organization. Handbook for guideline development. Chapter 10. Implementation and evaluation. Geneva, Switzerland: WHO; 2012.

[28] National Health and Medical Research Council (2011). Procedures and requirements for meeting the 2011 NHMRC Standard for Clinical Practice Guidelines.

[29] ACC/AHA Task Force on Practice Guidelines. Methodology manual and policies. Washington, DC: American College of Cardiology and American Heart Association; 2010.

[30] Canadian Task Force on Preventive Health Care. Procedure manual. Edmonton, AB: Canadian Task Force on Preventive Health Care; 2011.

[31] Krishnaswamy K (2008). Developing and implementing dietary guidelines in India. Asia Pac J Clin Nutr..

[32] Scottish Intercollegiate Guidelines Network (2008). A guideline developer’s handbook. Chapter 9. Presentation and dissemination. Chapter 10. Implementation.

[33] Davis D, Goldman J, Palda VA (2007). Handbook on clinical practice guidelines.

[34] Registered Nurses Association of Ontario (2012). Toolkit: implementation of best practice guidelines.

[35] Rosenfeld RM, Shiffman RN, Robertson P (2013). Clinical practice guideline development manual, third edition. Otolaryngol Head Neck Surg.

[36] Grimshaw JM, Schunemann HJ, Burgers J, Cruz A, Heffner J, Metersky M (2012). Disseminating and implementing guidelines. Article 13 in integrating and coordinating efforts in COPD guideline development. Proc Am Thorac Soc.

[37] Wilson KC, Irwin RS, File TM, Schunemann HJ, Guyatt GH, Rabe KF (2012). Reporting and publishing guidelines. Article 12 in integrating and coordinating efforts in COPD guideline development. Proc Am Thorac Soc.

[38] Haigekassa E, Ulikool T (2011). Estonian handbook for guidelines development.

[39] Van der Wees P, Mead J (2004). Framework for clinical guideline development in physiotherapy.

[40] Swiss Tropical and Public Health Institute (2011). Handbook for supporting the development of health system guidance.

[41] Graham R, Mancher M, Miller Wolman D, Greenfield S, Steinberg E (2011). Editors for the Committee on Standards for Developing Trustworthy Clinical Practice Guidelines, Institute of Medicine. Clinical practice guidelines we can trust.

[42] World Stroke Organization (2009). Clinical Practice Guideline Development Handbook for Stroke Care.

[43] Van der Wees PH, Hendrisk EJM, Custers JWH, Burgers JS, Dekker J, de Bie RA. Comparison of international guideline programs to evaluate and update the Dutch program for clinical guideline development in physical therapy. BMC Health Serv Res. 2007;7:191. 10.1186/1472-6963-7-191 PMC2228296 18036215

[44] Guidelines and Protocols Advisory Committee. Clinical Practice Guidelines Handbook. Victoria, BC: BC Guidelines, Guidelines and Protocols Advisory Committee; 2012.

[45] American Urological Association. Guidelines Department. Standard Operating Procedures. Linthicum, MD: American Urological Association, 2011.

[46] German Agency for Quality in Medicines. National Programme for Disease Management Guidelines—Method Report. Berlin, Germany: German Medical Association; 2010.

[47] European Society of Cardiology (2010). Recommendations for guideline production.

[48] Shekelle P, Woolf S, Grimshaw JM, Schunemann H, Eccles MP (2012). Developing clinical practice guidelines: reviewing, reporting, and publishing guidelines; updating guidelines; and the emerging issues of enhancing guideline implementability and accounting for comorbid conditions in guideline development. Implement Sci..

[49] American Academy of Neurology (2011). Clinical practice guidelines process manual.

[50] Kingston M, Radcliffe K, Daniels D, Fitzgerald M, Lazaro N, McCarthy G (2010). British association for sexual health and HIV: framework for guideline development and assessment. Int J STD AIDS..

[51] United States Preventative Task Force (2008). Procedure manual.

[52] American Society of Clinical Oncology (2011). Guidelines Methodology Manual.

[53] Hill J, Bullock I, Alderson P (2011). A summary of the methods that the national clinical guideline centre uses to produce clinical guidelines for the national institute for health and clinical excellence. Ann Intern Med..

[54] Iorio A, Ageno W, Cosmi B, Imberti D, Lussana F, Siragusa S (2009). Objectives and methodology: guidelines of the Italian society for haemostasis and thrombosis. Thromb Res..

[55] Dumonceau JM, Hassan C, Riphaus A, Ponchon T (2012). European society of gastrointestinal endoscopy guideline development policy. Endoscopy..

[56] American College of Rheumatology (2012). Policy and procedure manual for clinical practice guidelines.

[57] Qaseem A, Snow V, Owens DK, Shekelle P (2010). The development of clinical practice guidelines and guidance statements of the American College of Physicians: summary of methods. Ann Intern Med..

[58] Gupta S, Bhattacharyya O, Brouwers MC, Estey EA, Harrison MB, Hernandez P (2009). Canadian Thoracic Society: presenting a new process for clinical practice guideline production. Can Respir J.

[59] Zelman Lewis S, Diekemper R, Addrizzo DJ (2013). Methodology for development of guidelines for lung cancer, American College of Chest Physicians evidence-based clinical practice guidelines. Chest.

[60] Dougados M, Betteridge N, Burmester GR (2004). EULAR standardized operating procedures for the elaboration, evaluation, dissemination and implementation of recommendations endorsed by the EULAR standing committees. Ann Rheum Dis..

[61] Baker R, Camosso-Stefinovic J, Gillies C, Shaw EJ, Cheater F, Flottorp S (2010). Tailored interventions to overcome identified barriers to change: effects on professional practice and health care outcomes. Cochrane Database Syst Rev..

[62] Kajermo KN, Bostrom AM, Thompson DS, Hutchinson AM, Estabrooks CA, Wallin L (2010). The BARRIERS scale—the barriers to research utilization scale: a systematic review. Implement Sci..

[63] Traynor R, DeCorby K, Dobbins M (2014). Knowledge brokering in public health: a tale of two studies. Public Health..

[64] Jagosh J, Macaulay AC, Pluye P, Salsberg J, Bush PL, Henderson J (2012). Uncovering the benefits of participatory research: implications of a realist review for health research and practice. Milbank Q.

[65] Kothari A, Wathen CN (2013). A critical second look at integrated knowledge translation. Health Policy.

[66] Gagliardi AR, Brouwers MC, Bhattacharyya OK (2014). the Guideline Implementation Research and Application Network. A framework of the desirable features of guideline implementation tools (GItools): Delphi survey and assessment of GItools. Implement Sci..

[67] Giguere A, Legare F, Grimshaw J, Turcotte S, Fiander M, Grudniewicz A (2012). Printed educational materials: effects on professional practice and healthcare outcomes. Cochrane Database Syst Rev..

[68] Murthy L, Shepperd S, Clarke MJ, Garner SE, Lavis JN, Perrier L (2012). Interventions to improve the use of systematic reviews in decision-making by health system managers, policy makers and clinicians. Cochrane Database Syst Rev..

[69] McCormack L, Sheridan S, Lewis M, Boudewyns V, Melvin CL, Kistler C (2013). Communication and dissemination strategies to facilitate the use of health-related evidence.

[70] Forsetlund L, Bjorndal A, Rashidian A, Jamtvedt G, O’Brien MA, Wolf F (2009). Continuing education meetings and workshops: effects on professional practice and health care outcomes. Cochrane Database Syst Rev..

[71] Ivers N, Jamtvedt G, Flottorp S, Young JM, Odgaard-Jensen J, French SD (2012). Audit and feedback: effects on professional practice and healthcare outcomes. Cochrane Database Syst Rev..

[72] Long JC, Cunningham FC, Braithwaite J (2013). Bridges, brokers and boundary spanners in collaborative networks: a systematic review. BMC Health Serv Res..

[73] Meissner HI, Glasgow RE, Vinson CA, Chambers D, Brownson RC, Green LW (2013). The U.S. training institute for dissemination and implementation research in health. Implement Sci..

[74] Greenhalgh T, Russell J (2006). Promoting the skills of knowledge translation in an online Master of Science course in primary health care. J Cont Ed Health Prof..

[75] Straus SE, Brouwers M, Johnson D, Lavis JN, Legare F, Majumdar SR (2011). Core competencies in the science and practice of knowledge translation: description of a Canadian strategic training initiative. Implement Sci..

[76] Gagliardi AR, Webster F, Perrier L, Bell M, Straus S. Exploring mentorship as a strategy to build capacity for knowledge translation research and practice: a scoping systematic review. Implement Sci. 2014, in press. 10.1186/s13012-014-0122-z PMC4182766 25252966

