# Supplementary material for: Developing a checklist for guideline implementation planning: review and synthesis of guideline development and implementation advice
Source: Implement Sci. 2015 Feb 12;10:19. doi: 10.1186/s13012-015-0205-5 (PMC4329197; doi:10.1186/s13012-015-0205-5)
Supplement: Additional file 2: Table S2. — Search strategy (from MEDLINE). [file 13012_2015_205_MOESM2_ESM.doc]

Supplementary Table 2. Search strategy (from MEDLINE)

--------------------------------------------------------------------------------

1 use:.mp. (2853855)

2 implement:.mp. (175064)

3 (utilisation or utilization).mp. (84478)

4 adopt:.mp. (98148)

5 disseminat:.mp. (56966)

6 translat:.mp. (144860)

7 intervention.mp. (247746)

8 1 or 2 or 34 or 4 or 5 or 6 or 7 (3306683)

9 methods/ (4367)

10 manual.mp. (49098)

11 handbook.mp. (996)

12 guide.mp. (60444)

13 method:.mp. (2715428)

14 9 or 10 or 11 or 12 or 13 (2775600)

15 Practice Guidelines as Topic/st [Standards] (4926)

16 8 and 14 and 15 (1147)

17 limit 16 to (english language and yr="2004 - 2013") (782)

18 limit 17 to (comment or editorial or lectures or letter or news) (4)

19 17 not 18 (778)

20 limit 19 to practice guideline (3)

21 19 not 20 (775)

***************************
