# Supplementary material for: Developing a checklist for guideline implementation planning: review and synthesis of guideline development and implementation advice
Source: Implement Sci. 2015 Feb 12;10:19. doi: 10.1186/s13012-015-0205-5 (PMC4329197; doi:10.1186/s13012-015-0205-5)
Supplement: Additional file 3: Table S3. — Implementation guidance extracted from eligible resources. [file 13012_2015_205_MOESM3_ESM.doc]

Supplementary Table 3. Implementation guidance extracted from eligible resources

| Resource / Type of organization | Plan for implementation | Develop implementation products | Strategies for dissemination and implementation |
| --- | --- | --- | --- |
| Rosenfeld: American Academy of Otolaryngology--Head and Neck Surgery Foundation (35)  2013  Society/foundation | Page 25 Table 8  Discuss how the guideline will be disseminated, what barriers will be encountered and how they will be handled, and what supporting materials will be developed for implementation  S47  Staff who are unfamiliar with guideline content and not involved in its development use GLIA to appraise implementability | Page 40 Table 19  Consider the need for implementation materials to support the recommendation including brochures, teaching aids etc.  Page 52  Implementation products that have been used by guideline developers include short versions, patient (plain language) versions, recommendation summaries, algorithms, patient and caregiver resources  S46  Evaluation plans to assess impact of guideline on care and patient outcomes | Page 52   - Published on web site, presented at meetings, published in journal, press release, emails, podcasts, organization’s monthly bulletin and annual meeting. - Manuscripts can include links to illustrative videos and supporting materials online - Increase awareness by submitting to AHRQ National Guideline Clearinghouse and Guidelines International Network Library |
| Zelman-Lewis: American College of Chest Physicians (59)  2013  Society/foundation | --- | --- | Page 49  Journal publication |
| Grimshaw: American Thoracic Society (36)  2012  Society/foundation | Page 299  Identify barriers to change (structural, organizational, professional, patient) using observation, interviews, focus groups, or survey | Page 301 Table 3  Include tools that help users implement the recommendations: patient information, resource implications, implementation plan, implementation tools, evaluation tools | Page 299   - Select interventions based on barrier analysis using intervention mapping, or by input from stakeholders   Page 300 table 2   - Detailed table lists and describes seven approaches (printed educational material, educational meetings, educational outreach, local opinion leaders, audit & feedback, reminders and multi-faceted interventions) along with barriers they address, known effectiveness, and resource and practical considerations |

| Shekelle (48)  2012  Academic publication | --- | Page 4   - Resources to support implementation: patient information, resource implications, implementation plan, implementation tools, evaluation tools - Guideline summary for clinicians or policymakers, technical report for scientists, lay version for patients | Page 3  Journal and online publication |
| --- | --- | --- | --- |
| Wilson: American Thoracic Society (37)  2012  Society/foundation | Page 294  Describe barriers to use of the recommendations | Page 295   - Web repositories of the evidence that influences recommendations - Evidence summaries | Page 295  Duplicate publication in relevant journals, and publication in journals that are translated into multiple languages |
| Dumonceau: European Society of Gastrointestinal Endoscopy (55)  2012  Society/foundation | --- | --- | Page 629  Published on web site and in journal |
| Guidelines and Protocols Advisory Committee – British Columbia Guidelines (44)  2012  Government | --- | Page 13  Version for mobile devices, guideline summaries | Page 13  Published on web site and in journal, continuing professional development, conferences, email to guideline mailing list subscribers |
| National Institute for Health and Care Excellence (26)  2012  Government | Page 16   - Implementation team formed at the start of the guideline development process - During guideline planning an implementation advisor tracks implementation issues as they are revealed - Prepare an implementation support plan | Page 16  Each guideline accompanied by:   - Baseline assessment tool - Clinical audit tools - Costing report/template - Needs assessment may reveal the need for other educational tools   Chp 1.4.3  Four versions for different audiences:   - Full guideline with background, evidence and recommendations - Only recommendations - Online resource for healthcare and other professionals - Information for the public | Page 21   - Implementation tools are published at the same time as the guideline - Announced on web site and newsletter - Additional means of dissemination include conferences, workshops, meetings, journal publication - Partnerships with national organizations and networks further disseminate guidelines and tools |
| Registered Nurses Association of Ontario (34)  2012  Society/foundation | Page 76   - Identify implementation barriers and enablers - Enlist local champions and those with authority to supply resources - Select implementation strategies that align with available resources, and address identified barriers - Choose a starting point with a high chance of success to pilot your implementation strategy - Be prepared to monitor and adjust implementation strategies | --- | Page 142  Implementation strategy options:   - Audit & feedback - Educational materials - Educational meetings - Educational outreach visits - Organizational interventions (revision of professional roles or teams, leadership) - Opinion leaders - Mass media - Patient-mediated interventions |
| World Health Organization (27)  2012  Not for profit | Page 55   - Implementation should be considered from the beginning of guideline development - Engage regions, and national or subnational groups responsible for implementation in guideline development - To plan implementation:   - Analyze local needs/priorities   - Identify potential barriers   - Determine available resources   - Design an implementation strategy | Page 51, 52  Translated into six languages  Page 55  Derivative documents or tools can facilitate implementation, for example, slide set reflecting guideline content, “how to” manual, flow chart, decision aid or algorithm, fact sheets, quality indicators, checklists, application tools, templates, etc. These can be distributed with the guideline or developed by local implementers. | Page 51, 52  Disseminate via web site, mobile phone application, journal publication |
| American College of Rheumatology (56)  2012  Society/foundation | --- | --- | Page 21  Published on web site, published in journal |
| National Health and Medical Research Council (28)  2011  Government | Page 5  Implementation should be considered throughout the development of the guideline | Page 23   - Implementation plan - Resources to support implementation: summaries, tools, patient information, versions in different languages, evaluation measures, evaluation strategy | --- |

| American Society of Clinical Oncology (52)  2011  Society/foundation | --- | Section: Dissemination and Implementation  Patient materials, power point slide set, flow sheet or algorithm, decision aids | --- |
| --- | --- | --- | --- |
| American Urological Association (45)  2011  Society/foundation | --- | Page 11  Pocket guide, wall charts, patient information, performance measures | Page 4, 10, 11  Published on web site and in journal. Other means of dissemination include webinars, partnership with other organizations, and endorsement from other specialty societies |
| Haigekassa: Estonion Health Insurance Fund (38)  2011  Government | Page 4  Regularly assess performance of implementation plan  Page 56  Develop an implementation plan that includes:   - Identify potential barriers - Define implementation success criteria - Measure baseline data - Identify resources needed - Identify need for training/education - Consider how to communicate information to stakeholders - Use existing mechanisms such as clinical governance frameworks or performance management systems, and networks - Determine how implementation will be monitored - Determine clear roles and responsibilities for action items - Determine milestones with timescales for each implementation process | Page 55   - An algorithm will be included as a guideline appendix - Summary versions | Page 55  Available on web site |
| American Academy of Neurology  (49)  2011  Society/foundation | --- | Page 31  slide presentation, clinical case example, patient summary, clinician summary, algorithms | Page 31   - Publish in journal - Post on web site - Announce to members by email - Press release |

| Canadian Task Force on Preventive Health Care (30)  2011  Government | Page 43  Implementation planning questions:   - Who are the end-users of the guideline and who will be interested in its results? - What are the key messages for each of the end-users? - Who are the principal target audiences, organizations, and groups for each of these messages? - What are the barriers and facilitators to uptake of the guideline for each of these end-user groups? - What KT strategy will be used to facilitate uptake of the guideline?   Page 47, 48  Engage stakeholders at various points during the guideline development and implementation process:   - Identify and describes key project stakeholders - Engage stakeholders and sustain relationships - Develop stakeholder awareness through outreach and education at various increments throughout the development process - Monitor and evaluate ongoing stakeholder relationships on a guideline by guideline basis while continuing to seek out new engagement opportunities | Page 44, 45  Develop decision tools, mobile and electronic medical record applications:   - Tool planner is a member of the guideline development group - They conduct a needs assessment to determine the type of tools needed based on guideline scope and recommendations, identified knowledge gaps, or interviews or focus groups with guideline users - Research potential tool designs - Identify resources that will be needed - Draft tool presented to guideline development group for feedback - Feedback is used to refine the tool, several iterations may take place - Tool undergoes usability testing through interviews or focus groups with clinicians or patients - Feedback is used to refine the tool - Final version is reviewed by guideline development group | Page 48  Published in journals, presented at conferences, mass media campaigns |
| --- | --- | --- | --- |
| Hill: National Clinical Guideline Centre for NICE (53)  2011  Government | --- | Page 757  Costing tools (national cost report, costing spreadsheet), audit criteria, slide set, implementation suggestions, educational tools | --- |

| Graham: Institute of Medicine (41)  2011  Government | Page 171   - Guideline developers should structure the format, vocabulary and content of guidelines to facilitate integration into computer decision support systems (CDSS) - Guideline developers should collaborate to more readily integrate guidelines into CDSS | --- | Page 149, 150  Options for implementation strategies:   - Clinical reminders - Quick reference guides/summaries - Mass media - Printed educational material - Educational meetings - Opinion leaders - Academic detailing - Audit & feedback - Pay for performance - Computer decision support systems |
| --- | --- | --- | --- |
| Swiss Tropical and Public Health Institute (40)  2011  Government | Page 101  To create summary versions or other products include end-users on the production team and determine the best means (format, content, dissemination and implementation strategies) to reach target audiences | --- | Page 105  Options for implementation include:  (noted inconsistent effectiveness)  Educational material  Educational meetings  Educational outreach  Opinion leaders  Patient-directed interventions  Audit & feedback  Reminders  Organizational interventions  Structural interventions  Regulatory interventions |
| Kingston: British Association for Sexual Health and HIV (50)  2010  Society/foundation | --- | Page 454  Patient leaflets, quick reference guide of key recommendations, clinical care algorithm, audit measures | --- |
| Qaseem: American College of Physicians (57)  2010  Society/foundation | --- | --- | Page 199  Published on web site and in journal |
| German Agency for Quality in Medicines (46)  2010  Society/foundation | --- | Page 12  Short version, pocket guide, patient version, long version with references | Page 12  Published on web site and in journal. Other means include conferences and internet-based modules for accredited continuing medical education |
| American College of Cardiology / American Heart Association (29)  2010  Society/foundation | --- | Page 59  Full and summary version and pocket guide | Page 59  Published on web site and in journal publication |
| European Society of Cardiology (47)  2010  Society/foundation | --- | Page 4  Pocket version, slide sets  Page 9  Summary versions, posters, slide-sets, CD-ROMs and organization of meetings  Page 13  Full, summary and mobile applications | Page 13  Published on web site, full text published in journal, accompanied by press release |
| Gupta: Canadian Thoracic Society (58)  2009  Society/foundation | --- | --- | Page e67  Active knowledge translation strategies will be launched and all new guideline materials will be posted on the web site |
| Iorio: Italian Society for Haemostasis and Thrombosis (54)  2009  Society/foundation | --- | Page 5  Each guideline includes a strategy for its implementation and lists barriers to its application that may be structural, organizational, technical or professional | --- |
| World Stroke Organization (42)  2009  Society/foundation | Page 11  An implementation plan should be developed simultaneous to guideline development. | Page 11  Summary document, summary slides | Page 11  Publish in relevant journal, professional educational, audit & feedback, accreditation |
| Krishnaswamy (31)  2008  Academic | Page 66   - For successful implementation of guidelines, political/bureaucratic commitment is essential - Adopt partnerships with experts from multiple disciplines e.g. opinion leaders, educators, government agencies, consumers, specialists, media personnel   Page 67  Implementation should consider the country's existing practices, as well as the geographical, social, political, economic and cultural factors. | Page 68   - Booklets, leaflets, attractive posters and folders with emphasis on pictorial representation - Multi-media communication package | Page 66   - Workshops and meetings - Marketing strategies - Interpersonal communication and professional societies   Page 68   - Traditional arts like folk media and street plays - Print media - Press conferences |

| Scottish Intercollegiate Guidelines Network (32)  2008  Government | Page 47   - Assemble an implementation planning group including interprofessional stakeholders, both a leader and a facilitator, and patient groups - Audit current practice   Page 43  Identify barriers to implementation:   - Structural (financial disincentives) - Organisational (skill mix, facilities or equipment) - Peer group (patterns of practice) - Individual (knowledge attitudes, skills) - Professional-patient interaction (communication, information processing)   Page 47   - Prepare target users through communication, assess patient preferences through surveys, and acquire new equipment or change forms - Decide which implementation techniques to use based on evidence of effectiveness and barriers identified - Prepare an action plan that names responsible person, time scale and contingency plans - Evaluate progress and modify plan as necessary | Page 41  The guideline should include a quick reference guide of key recommendations, key points for audit, suggested outcome measures, and information for patients and carers | Page 45, 46   - Web site is used to disseminate guidelines and related products. - Implementation is a local responsibility and should include active educational intervention, and patient-specific reminders relating directly to professional activity. - Effective implementation strategies include reminders, educational outreach, interactive educational workshops, multi-faceted interventions. - Those of variable effectiveness include audit & feedback, consensus conferences, opinion leaders, patient mediated interventions, financial incentives, written materials, mass media |
| --- | --- | --- | --- |
| United States Preventative Services Task Force (51)  2008  Government | --- | Page 18  Full and summary version | Page 18  On web site, published in journals |

| Davis: Canadian Medical Association (33)  2007  Society/foundation | Page 17, 18   - Plan implementation for individual recommendations rather than the entire guideline so that the effort is manageable. - Form an implementation working group that includes those who will be affected by the guideline and knowledge translation experts   Page 18 Table 3.1   - Use surveys, focus groups, interviews, observation or case studies to identify barriers of adoption (professionals, patients, organization, economics, policy - Detailed table of questions and issues to assess contextual barriers of change   Page 24  Identify groups, agencies or organization with whom to partner for implementation | --- | Page 23  Select implementation strategies based on barriers (mentions that there is no proven way to do this)  Page 20 Table 3.2  Detailed table lists several professional (educational materials and meetings, educational outreach or academic detailing, audit & feedback, reminders, local opinion leaders), patient (educational materials, mass media campaign, reminders, decision-support tools) and organizational or regulatory strategies (changes to health care teams, electronic decision support, audit & feedback, administrative procedures or policies, formularies, financial incentives or penalties, regulated practices) for implementing guidelines, offers a brief description of each, and references a study in which the strategy was used |
| --- | --- | --- | --- |
| Van der Wees: Royal Dutch Society for Physical Therapy (43)  2007  Society/foundation | --- | Page 5   - Summary and patient versions - English translation - Implementation plan | Page 5  Published on web site or in peer-reviewed journal |
| Van der Wees: European Region of the World Federation for Physical Therapy (39)  2004  Society/foundation | Page 12  Relevant factors for the successful implementation of a guideline can be identified during the development of guidelines | Page 12  Audit packs | Page 12  Requires a rigorous strategy with different activities that may include: publish guideline, organize workshops, present at conferences, continuing professional education, reminders |
| Dougados: European League Against Rheumatism (60)  2004  Society/foundation | --- | --- | Page 1175  Publish in journal, present at meetings. Other techniques have been proposed including opinion leaders, academic detailing, audit & feedback, continuing medical education, reminders |
